# Case of Tetanus from a Punctured Wound in the Heel Cured by the Tincture of Cantharides: Communicated in a Letter from Dr. Samuel Brown, of Lexington (Kentucky), to Dr. Mitchill, of New York

**Published:** 1808-05

**Authors:** 


### CASE *of* TETANUS *from a* PUNCTURED WOUND *in the* HEEL *cured by the* TINCTURE *of* CANTHARIDES: *Communicated in a Letter from Dr.* SAMUEL BROWN, *of Lexington (Kentucky), to Dr.* MITCHILL, *of New York.*

ON Wednesday, the 28th of October, 1800, I visited Miss P— D—, a young lady aged nineteen. On Monday night, previous to my visit, she, unfortunately, set her foot upon anail, which, entering the middle of the heel, penetrated quite to the os calcis At first she complained of a numbness rather than pain; but in the course of half an hour the pain became tormenting in the wound, from which, however, it soon removed to the sides of the ancle, leaving the puncture perfectly free from any uneasiness. During the night she continued restless, though the pain, at intervals, considerably abated. On Tuesday evening all the symptoms became more violent. The wound discharged nothing except a drop or two of blood and serum; the pain became more acute in the ancle, and extended to the knee, hip and joints of the back. In the night she had many twitches of the nerves, and spasms of the muscles of the arms, and those which retract the head. Before the morning there was so great a degree of rigidity in the back of the neck, that the relations were at no loss with regard to the nature of her complaint. A physician was sent for, who unluckily, was at a horse-race, and declined to visit her.

her. He, however, sent a few anodyne pills, which she took without any material advantage. It was twelve o'clock on Wednesday night when I arrived. I found the patient in extreme agony, with spasms in her back and neck, and acute pains in her ancle, knee, and hip joint. On examining the puncture, not the slightest appearance of inflammation could be discerned; the heel was preternaturally cold, the skin was pale, and nothing but blood and serum could be pressed from the wound. I introduced my probe, and was greatly surprised to find that she was scarcely sensible of pain from a very harsh examination of the direction and extent of the puncture. At length, however, the point of my probe touched a nerve or ligament, which appeared to possess extreme sensibility. Having fixed the probe firmly against the os calcis, I took a scalpel, and cut down the side of the probe through the ligaments quite to the bone, in a transverse direction with regard to the foot. Immediately on making this incision, the whole of her complaints seemed to be removed, and I flattered myself, that I should be able, by the usual remedies, to prevent a recurrence of the spasms. But, anxious more effectually to destroy the injured nerve, and to excite inflammation in the part, I washed the wound with spirits of turpentine; and, having made a dossil of lint, I covered it with powdered sulphate of copper and spirits of turpentine, and pressed it down to the bottom of the incision. This application gave severe pain; but the patient assuring me, that it was much more tolerable than the spasms, I suffered it to remain. I now gave her a very large dose of laudanum, which I directed to be repeated every four hours—I likewise laid a large blister on the ancle, where the pain had been always very distressing some time before it extended to the body. Warm poultices were kept to the heel. I ordered two grains of calomel every hour when she was awake, and had ℥ i of mercurial ointment rubbed into the legs, arms, and back—I likewise endeavoured to hasten salivation, by rubbing calomel on the gums. She slept about two hours, and continued tolerably easy until twelve or one o'clock on Tuesday, when her spasms returned—I was again sent for, and arrived about dark. All the symptoms were then greatly aggravated; the spasms more violent than I had ever seen them; they were more general—her jaws were stiff, her deglutition difficult, and the stridor dentium so great as to be heard in a very distinct manner all over the house. Recollecting the cases which occurred to Mr. Dallas, and his friends, in the island of Carcracow, as as published in the Edinburgh Medical Annals, from which, it appears, that not one person in fifty who is affected with tetanic symptoms, from ths læsion of a nerve, recovered, notwithstanding the most approved modern remedies were vigorously employed, I resolved to give my patient a chance for life, by attempting to relieve her by a new mode of treatment.

The case appeared so hopeless, and death so near, that I thought the "*anceps remedium*" perfectly justifiable. Having, in vain, endeavoured to subdue the spasms, by antispasmodics, to produce salivation by the liberal exhibition of mercury, and to excite inflammation in the wound by the strongest stimulants, I determined to make trial of wine and bark, which had proved so efficacious in the hands of Dr. Rush. These were immediately rejected by the stomach. The blister, after remaining on the ancle, from Wednesday until Friday night, produced very little effect on the skin.

By this time ℥ ij of mercurial ointment had been rubbed in, xxij grains of calomel taken in form of pills, and ℈ i. rubbed on the gums. I now despaired of being able to excite ptyalism early enough to prevent the fatal termination of the disease. I therefore directed fifteen drops of the Tinctura Cantharid. in a cup of tea, to be taken every hour, until the symptoms of inflammation in the bowels forbid their further exhibition. I now removed the dressings from the wound, and filled it with the root of the phytolacca decandra (or poke-root), which produced very great uneasiness for two or three hours. The hot poultices were continued, together with the use of injections, which had been found necessary during the whole course of the disorder.

After the patient had taken ʒjß of the tincture of cantharides, she was seized with a sensation of heat and burning in the stomach, which, becoming more and more severe, extended through the whole course of the alimentary canal, and produced a number of stools, mixed with blood and mucus. The inflammation of the stomach occasioned vomiting, and the acute pains in her bowels resembled those which are felt in the most violent attacks of dysentery. *Immediately on the occurrence of these symptoms, all the tetanic affections disappeared, and never returned.* In order to mitigate the disease which I had produced in the alimentary canal, I gave large quantities of mucilaginous drinks, and anodyne enemata, which afforded considerable ease. Fortunately, however, the day after this inflammatory tory tory state of the bowels came on, the mercury, which before seemed quite inert, began to show its effect on the salivary glands, and (as we have often seen in dysentery) removed the disease of the intestines. Some slight affection of the kidneys appeared, with bloody urine, but soon left the patient without any complaint, except a very moderate salivation, which continued for about two weeks. The wound began to form pus eight days after the accident, and was kept open several weeks, from an apprehension, that if it closed, the spasms might return.

This case, which terminated so happily, is not communicated to the public as the foundation of any new favourite theory. The writer is sufficiently aware of the danger of deducing general inferences from solitary facts. Disappointed in his expectations of relieving his patient by the fashionable remedies, his practice was the result of that necessity which occasionally gives birth to the most useful discoveries. He thought it his duty to communicate it with simplicity and candour, and hopes that those who practise medicine in climates where tetanus is a common disease, will strive to lessen its mortality, by giving cantharides a fair trial. It is well known that physicians are often under the necessity of combating one disease by creating another, and although it must be confessed that the inflammation of the stomach and bowels is attended with great danger, no practitioner will hesitate to affirm that tetanus is still more dangerous.

